# Succinate supplementation improves metabolic performance of mixed glial cell cultures with mitochondrial dysfunction

**DOI:** 10.1038/s41598-017-01149-w

**Published:** 2017-04-21

**Authors:** Susan Giorgi-Coll, Ana I. Amaral, Peter J. A. Hutchinson, Mark R. Kotter, Keri L. H. Carpenter

**Affiliations:** 1 Division of Neurosurgery, Department of Clinical Neurosciences, University of Cambridge, Box 167, Cambridge Biomedical Campus, CB2 0QQ UK; 2 Anne McLaren Laboratory, Wellcome Trust MRC Cambridge Stem Cell Institute and Department of Clinical Neurosciences, University of Cambridge, West Forvie Building, Robinson Way, Cambridge, CB2 0SZ UK

## Abstract

Mitochondrial dysfunction, the inability to efficiently utilise metabolic fuels and oxygen, contributes to pathological changes following traumatic spinal cord or traumatic brain injury (TBI). In the present study, we tested the hypothesis that succinate supplementation can improve cellular energy state under metabolically stressed conditions in a robust, reductionist *in vitro* model of mitochondrial dysfunction in which primary mixed glial cultures (astrocytes, microglia and oligodendrocytes) were exposed to the mitochondrial complex I inhibitor rotenone. Cellular response was determined by measuring intracellular ATP, extracellular metabolites (glucose, lactate, pyruvate), and oxygen consumption rate (OCR). Rotenone produced no significant changes in glial ATP levels. However, it induced metabolic deficits as evidenced by lactate/pyruvate ratio (LPR) elevation (a clinically-established biomarker for poor outcome in TBI) and decrease in OCR. Succinate addition partially ameliorated these metabolic deficits. We conclude that succinate can improve glial oxidative metabolism, consistent our previous findings in TBI patients’ brains. The mixed glial cellular model may be useful in developing therapeutic strategies for conditions involving mitochondrial dysfunction, such as TBI.

## Introduction

Following traumatic injury to the spinal cord or brain, a complex combination of pathological processes develop, in which cerebral energy perturbations and cellular metabolism play a key role^1–5^. Despite modern advances in acute neurocritical care, many traumatic brain injury (TBI) or spinal cord injury (SCI) patients who survive the injury experience long-term disability. Greater understanding of the pathophysiology of the injured central nervous system (CNS) is needed to improve early neurocritical care. Previous research has suggested that ‘mitochondrial dysfunction’, where the brain is unable to efficiently utilise metabolic fuels and oxygen despite adequate provision, may underlie damaging metabolic disturbances, which progress after injury. Notably, high brain extracellular lactate/pyruvate ratio (LPR), suggestive of high glycolytic activity and reduced mitochondrial function, correlates with unfavourable clinical outcome^4^.

Early studies of cellular metabolism in the injured brain focussed on ischaemia, which has been minimised in the modern clinical care setting by maintaining adequate cerebral perfusion, as well as intracranial pressure below a critical threshold^6^. In the absence of ischaemia, mitochondrial dysfunction is thought to be responsible for energy perturbations in the brain after injury^5, 7^. A reduced Ca^2+^ uptake state after TBI is thought to be relevant here, as this results in an increase in Ca^2+^ in the cytosol, which is taken up by mitochondria with great efficiency at the expense of membrane potential. As a consequence, a condition termed Ca^2+^ overload develops, which is characterised by reduced mitochondrial dehydrogenase activity and subsequent suboptimal mitochondrial-driven metabolism^8–10^. In addition, the production and activity of reactive oxygen species (ROS) is thought to play a key role, affecting mitochondrial membrane potential and damaging membrane-bound components^10^.

Succinate plays a pivotal role in oxidative metabolism. Succinate is a tricarboxylic acid (TCA) cycle intermediate that interacts directly with the mitochondrial electron transport chain (ETC), enabling a ‘shortcut’ route to ATP production via oxidative metabolism. This has been suggested as a potential therapeutic strategy for TBI^11^. Succinate is converted to fumarate in the TCA cycle by complex II (succinate dehydrogenase (SDH)) of the ETC on the mitochondrial inner membrane^12, 13^. The remainder of the TCA cycle is driven by soluble mitochondrial enzymes, resulting in the production of NADH which interacts with complex I of the ETC^12, 13^. This in turn drives the ETC sequentially from complex I to Coenzyme Q (CoQ) through to culmination at complex IV, missing complex II^12, 13^. Succinate, contrastingly, misses complex I and causes the ETC to run sequentially from complex II all the way through to complex IV (converting oxygen to water)^12, 13^.

Complex I of the ETC is known to be particularly vulnerable to dysfunction or damage, especially in the presence of ROS^14^. In bypassing complex I, succinate can provide a potential fuel source for compromised mitochondria in order to produce ATP and maintain membrane potential. Preventing ATP levels from falling to critically low levels is crucial in blocking the activation of cell death pathways that would result in loss of brain tissue. The strategy of ‘bypassing’ defective ETC components was first reported by Eleff *et al*., who demonstrated that muscle ATP synthesis in a patient with a genetically defective form of complex III could be maintained by supplementation with vitamins C and K^15^. In other studies, infusion of succinate, which bypasses complex I, prevented decline in liver ATP levels and prolonged survival in cases of experimental sepsis, a pathology involving energy dysfunction^14, 16^. In cultured fibroblasts from a patient with Leigh syndrome due to a genetic mutation in complex I, succinate prodrugs added *in vitro* to the cells improved mitochondrial respiration^17^.

The aim of the present study was to investigate whether succinate supplementation can rescue the energy state in rat mixed glial cells under experimental metabolic stress conditions using a defined inhibitor of mitochondrial function. Glia are especially relevant as clinical monitoring of TBI brain is performed via microdialysis catheters that are predominantly situated in white matter. In the present *in vitro* study, mixed glial cell cultures were isolated from neonatal Sprague Dawley rats. The cultured cells were exposed to rotenone, which is an inhibitor of complex I of the mitochondrial ETC, for periods of up to 24 h to induce conditions of metabolic stress, in the presence or absence of disodium succinate. Cellular response was determined by the measurement of intracellular ATP, extracellular metabolites of clinical relevance (glucose, lactate, pyruvate), and the oxygen consumption rate (OCR) and extracellular acidification rate (ECAR). The mixed glial cultures were characterised by immunocytochemistry.

## Results

### Characterisation of mixed glial cultures

**Figure 1:**
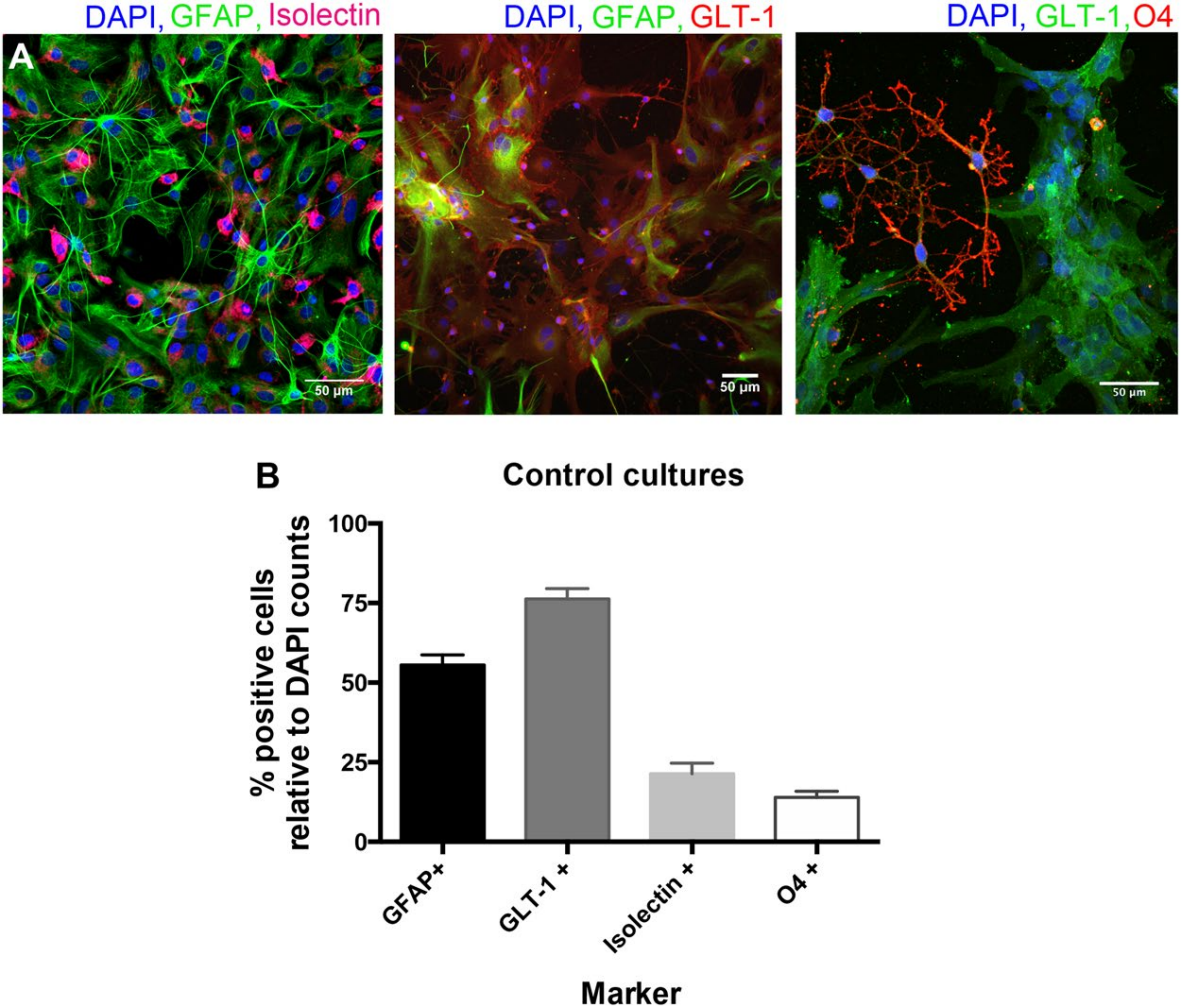
Characterisation of the cell populations present in the mixed glial cell cultures. Cells were stained for GFAP and GLT-1 (astrocyte markers), Isolectin (microglia marker) and O4 (oligodendrocyte marker). Representative images are shown in (**A**); total cell counts with the distribution of cell types is shown in (**B**).

We first characterised our primary rat mixed (glial) cell culture model using immunocytochemistry. GFAP and GLT-1 were used as astrocyte markers, Isolectin as a microglia marker, and O4 as an oligodendrocyte markers (Fig. 1A). The cultures comprised predominantly of astrocytes (at around 55% of GFAP positive cells and 76% of GLT-1 positive cells), as well as microglia (approximately 21% of Isolectin-positive cells) and oligodendrocytes (approximately 14% of O4-positive cells) (Fig. 1B).

### Mimicking metabolic stress in mixed glia cultures

To mimic metabolic features of TBI *in vitro*, metabolic stress was induced by treatment of mixed glia cultures with rotenone, an inhibitor of complex I of the ETC. We first optimised our model by assessing the effects of varying concentrations of rotenone (up to 10 µM), which all resulted in a significant decrease of basal OCR to approx. 30% of the control rate. In contrast, levels of intracellular ATP, measured after a 24 h incubation with increasing concentrations of rotenone remained comparable (data not shown). All subsequent experiments were conducted using 0.1 µM or 0.5 µM rotenone.

### Effect of succinate on mitochondrial activity

**Figure 2:**
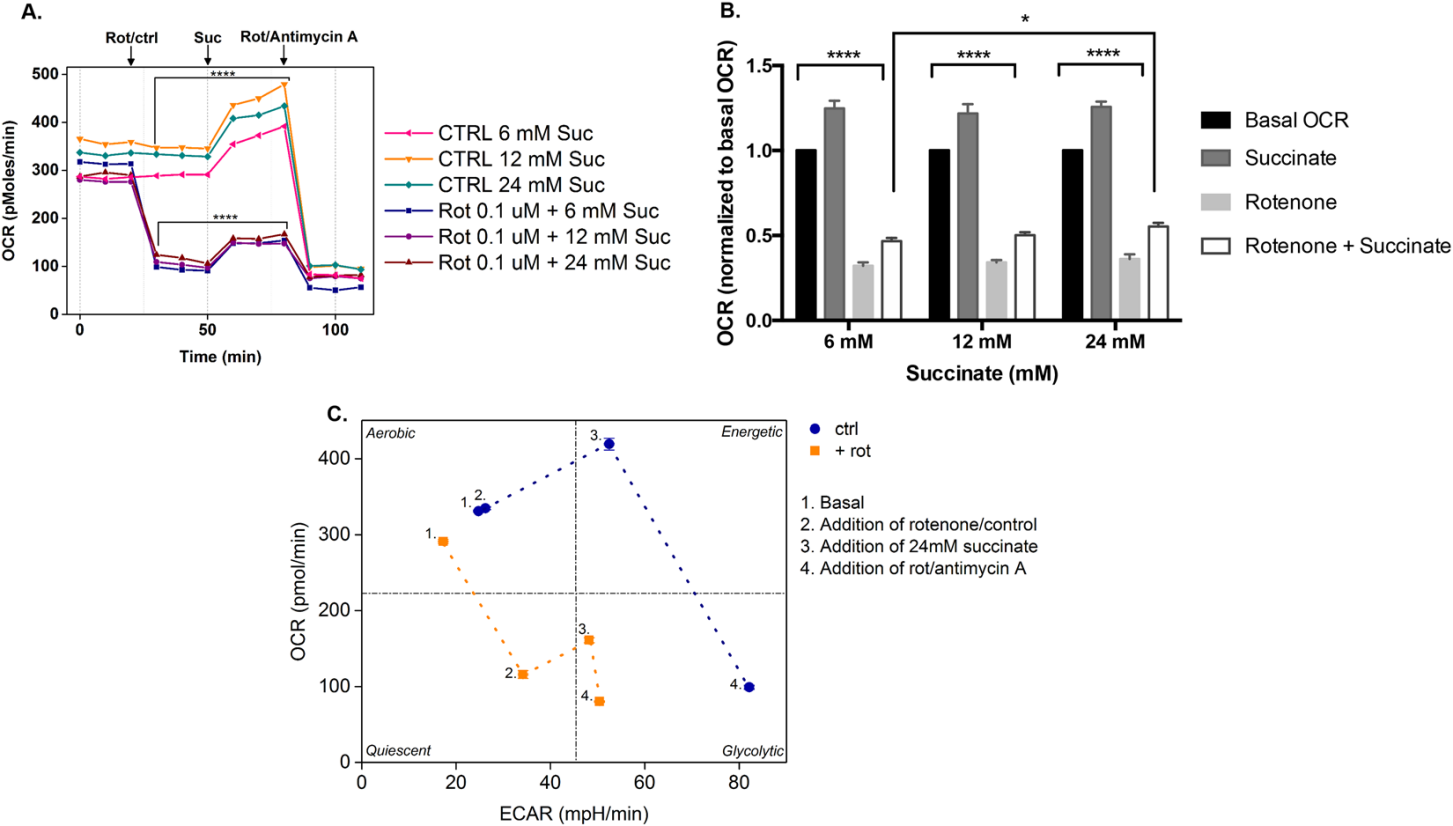
Effect of rotenone and succinate on oxygen consumption rate of mixed glia cultures. Oxygen consumption rate (OCR) was measured at 10 min intervals using a Seahorse Extracellular Flux XF24 analyser, in response to treatment with 0.1 µM rotenone (Rot; mitochondrial complex I inhibitor) (or regular assay medium in the control wells) followed by addition of succinate (Suc; 6, 12, 24 mM) and, finally, after oxidative metabolism is blocked by the addition of rotenone plus antimycin A (mitochondrial complex III inhibitor) in the final three measurements. (**A**) OCR profile over the course of the experiment; (**B**) Highlight of the effect of succinate on basal OCR under control conditions (addition of assay medium) and in the presence of succinate alone, 0.1 µM rotenone alone, or after the sequential addition of rotenone and different concentrations of succinate, as indicated. Statistically significant differences in OCR were observed between control samples and cells treated with succinate, rotenone, or succinate + rotenone for all succinate concentrations tested; furthermore, when comparing the ability of succinate to increase OCR after rotenone treatment, its effect at 24 mM was significantly higher than that induced by 6 mM. A 2-way ANOVA followed by Tukey’s multiple comparisons test was used and simple effects within columns and rows were analysed. Statistical significance is denoted as follows: P ≤ 0.05 (*) and P ≤ 0.0001 (****). (**C**) Metabolic phenotype plot of OCR vs. extracellular acidification rate (ECAR). Data are from the same wells as in (**A**). Blue circle symbols indicate control wells: (1) basal conditions, (2) addition of control assay medium, (3) addition of 24 mM succinate, (4) addition of rotenone plus antimycin A. Orange square symbols indicate experimental wells: (1) basal conditions, (2) addition of 0.1 µM rotenone, (3) addition of 24 mM succinate, (4) addition of rotenone plus antimycin A. Units of OCR are picomol/min and ECAR milli-pH units/min; values are mean (n ≥ 3) ± standard error of the mean (s.e.m).

Treatment with 0.1 µM rotenone induced an immediate reduction of OCR to approximately 1/3 of levels in controls (Fig. 2A). Following the addition of 6, 12 or 24 mM succinate, OCR rate increased in both control wells and in cells treated with rotenone (n ≥ 3 wells per condition; Fig. 2). To confirm mitochondrial viability, the final step involved complete inhibition of oxidative metabolism by combinatorial treatment with rotenone/antimycin. Figure 2B represents the observed improvements of mitochondrial activity induced by succinate, which resulted in an approximate 20% increase of OCR in rotenone-treated as well as control cells. The corresponding metabolic phenotype plot (Fig. 2C) also indicates shifts of the rotenone-treated cells and control cells to more energetic states as a result of addition of succinate. OCR indicates mitochondrial respiration, while ECAR is an indicator of glycolysis. Another source of extracellular acidification is CO_2_ production by mitochondria. For further explanation of OCR and ECAR see Divarakuni *et al*.^18^.

### Effect of succinate on clinically relevant metabolite levels

**Figure 3:**
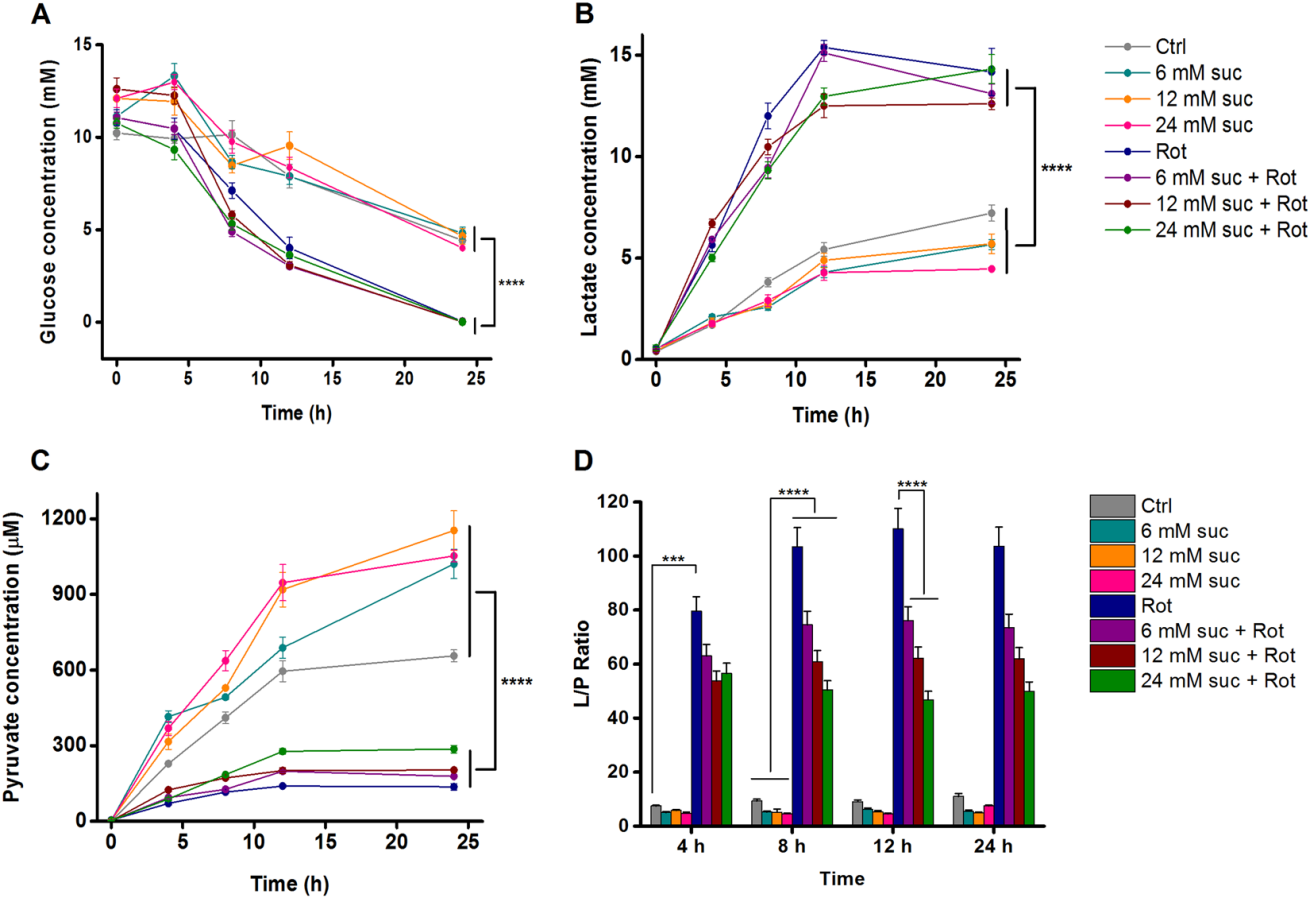
Dynamic changes in extracellular metabolites after treatment with rotenone and succinate in mixed glia cultures. Samples of medium were collected at 4 h intervals during 24 h and extracellular metabolites were measured using a clinical microdialysis analyser (ISCUSflex) in response to 0.5 µM rotenone treatment, with or without succinate (6, 12, 24 mM) supplementation. The average concentrations of the metabolites glucose, lactate and pyruvate are shown in (**A**), (**B**), and (**C**), respectively. The lactate/pyruvate ratio (LPR) is shown in (D). Values are the mean of n = 3 (each measured in duplicate), ±standard error of the mean (s.e.m.). A total of 3 independent samples are shown from one representative experiment (further results not shown). Statistically significant differences were observed between samples treated with rotenone and controls (1-way ANOVA), and between samples with or without succinate (paired sample t-test). Statistical significance is denoted as follows: P ≤ 0.001 (***) and P ≤ 0.0001 (****).

The OCR experiments demonstrated an instantaneous effect of rotenone on mitochondrial function. To evaluate the dynamic response of mixed glial cultures to 0.5 µM rotenone alone, or in the presence of 6, 12 or 24 mM succinate, a time-course experiment was conducted. Changes in extracellular metabolites were monitored every 4 hours over the course of 24 h (n = 3 wells per condition; Fig. 3). Rotenone induced accelerated glucose depletion in the medium (Fig. 3A). This effect did not change in the presence of succinate. Rotenone treatment was also associated with higher concentrations of lactate in supernatants (Fig. 3B). In contrast, pyruvate levels were significantly lower in the wells treated with rotenone (Fig. 3C). The presence of succinate in the absence of rotenone resulted in increased pyruvate levels compared to control wells, suggesting an enhancement of mitochondrial activity by succinate. The most striking response was seen in the LPR, which is clinically considered the most reliable measure of metabolic stress within the injured brain (Fig. 3D). The LPR increased significantly with time in the presence of rotenone, showing the deterioration of the cells’ metabolic performance (Fig. 3D). On the other hand, succinate decreased the LPR in a concentration-dependent manner and this effect stayed constant with time, showing a sustained improvement of metabolic function, tested for up to 24 h.

### Cell viability and expression of cellular markers

**Figure 4:**
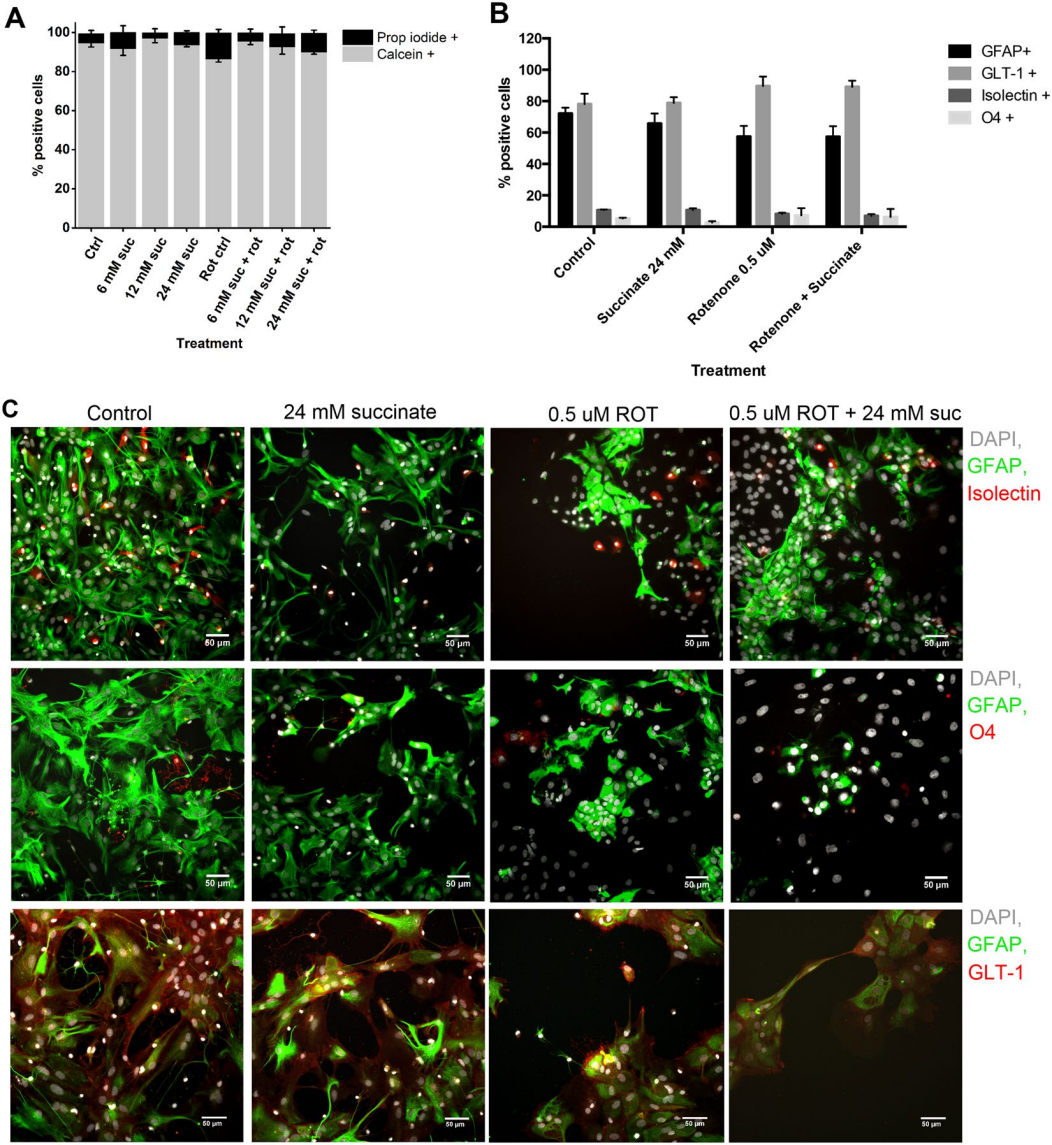
Effect of rotenone and succinate on cell viability and expression of cellular markers. (**A**) Percent of total cells stained with propidium iodide (dead cells) and calcein (viable cells) after 24 h of treatment with succinate and/or 0.5 µM rotenone v. control conditions. (**B**) Percent of total cells expressing the different cellular markers (GFAP, GLT-1, isolectin and O4) after 24 h of succinate treatment, with or without 0.5 µM rotenone. All values in A and B are mean ± standard error of the mean (s.e.m.) of >500 cells analysed (n = 6 individual wells from 2 independent experiments). The expression of cellular markers did not change significantly with rotenone or succinate treatment. (**C**) Images of the mixed glial cells stained with the markers DAPI (grey), GFAP (green), GLT-1 (red), isolectin (red) and O4 (red) after 24 h of succinate treatment, with or without 0.5 µM rotenone.

Cell death after treatment with rotenone and succinate for 24 h was determined by staining live cultures with propidium iodide (which labels the nuclei of dead cells), and calcein-AM (which stains viable cells in green after being converted to calcein). Figure 4A illustrates that the addition of 0.5 µM rotenone resulted in limited propidium iodide labelling of cells, comparable to control conditions and succinate-treated cells (n = 6 individual wells from 2 independent experiments). To assess the effects of rotenone and succinate on specific cell types, cells were labelled for GFAP and GLT-1 (astrocytes), isolectin (microglia), and O4 (oligodendrocyte precursor cells using immunocytochemistry (Fig. 4B,C). Rotenone treatment did not result in statistically significant differences amongst cell types (Fig. 4C).

## Discussion

Mitochondrial dysfunction, where the brain is unable to utilise metabolic fuels and oxygen despite adequate provision, is thought to play a major role in the energy perturbations that occur following TBI. The aim of the present study was to investigate whether succinate treatment could improve cellular metabolism in a reductionist metabolic stress model, akin to the metabolic alterations observed in TBI patients after succinate treatment^11^.

To model mitochondrial dysfunction in brain, primary rat mixed glial cell cultures were treated with rotenone to induce metabolic stress. The cultures consisted predominantly of astrocytes, next to oligodendrocyte lineage cells and microglia. Our model enabled studying mitochondrial dysfunction, induced by rotenone-mediated inhibition of complex I. This is specifically relevant to brain injury without ischaemia, where the susceptibility of complex I in the mitochondrial transport chain to sustain damage is thought to play a major role^10^. Our reductionist approach enabled assessment of the remaining metabolic capacity, and the possible metabolic “rescue”-type function of succinate administration. We specifically assessed changes in oxygen consumption rate (OCR), intracellular ATP, and in the levels of the extracellular metabolites glucose, lactate, pyruvate, and lactate in response to rotenone treatment and after succinate administration.

We observed a significant reduction in OCR following rotenone treatment at concentrations ranging from 0.1 to 10 µM. This confirmed significant mitochondria dysfunction, even at low concentrations of rotenone. Glucose, pyruvate and lactate are metabolites regularly measured in the brain extracellular fluid of TBI patients in a clinical setting to establish phenotypes that can be correlated with a favourable or unfavourable outcome. Mixed glial cultures exposed to rotenone exhibited abnormalities in glucose, lactate and pyruvate levels which represent the main metabolic features previously described in TBI patients. A cellular model of this type has not previously been described within the field of brain metabolism and could be very valuable to the study of metabolic interactions not just in an injured state, but also within a healthy and functioning glial cell system.

Our initial experiments established that cultures were able to partially compensate rotenone-induced metabolic stress and maintain ATP levels in the 24 h time-frame of the experiments. This may result from the capacity of glial cells to increase glycolysis or autophagy for producing ATP^19^. In fact, it has been demonstrated that astrocytes can generate lactate from glycogen via glycolysis under conditions of metabolic activation, including hypoglycemia and increased neuronal activity^20–22^.

In a clinical setting, increased extracellular (microdialysate) lactate versus pyruvate, expressed in an increased LPR can be indicative of impaired oxidative metabolism in TBI patients^3, 5, 7, 23^. Hillered *et al*. identified two possible circumstances which can lead to LPR elevation clinically^23^. The first is classical ischaemic metabolic state characterised by increased lactate due to anaerobic metabolism, which is accompanied by markedly low pyruvate. In the second, pyruvate is diminished in the absence of ischaemia, indicating metabolic disturbance. The latter is considered as associated with mitochondrial dysfunction. Recently Nordström *et al*. have highlighted that transitions can occur within-patient between ischaemia-pattern and mitochondrial dysfunction-pattern (and vice versa), with time post-injury, and that in the transition from ischaemia-pattern to mitochondrial dysfunction, pyruvate can rise with time^7^. In patients with TBI, the mitochondrial dysfunction pattern of LPR elevation is seen more often than ischaemic pattern^5, 7^. In our cell culture model, LPR elevation is a result of rotenone treatment that inhibits mitochondrial ETC complex 1, producing significantly elevated lactate levels, as well as reduced pyruvate levels, which contributed to an increase in the LPR in rotenone-treated cells. Furthermore, rotenone treated cells consumed more glucose compared to controls over the course of 24 h (Fig. 4A). This likely reflects the capacity of the glial cells to increase their metabolism of glucose via glycolysis to compensate for energy deficits in mitochondrial oxidative metabolism.

The main goal of the present work was to assess to what extent the administration of succinate could improve mitochondria function after rotenone treatment. The addition of succinate (with or without rotenone treatment) improved OCR by approximately 20% (Fig. 2). Since the same extent of increase in OCR was observed for healthy control cells (not pre-treated with rotenone), we hypothesize that there might be a limiting factor regarding the ability of succinate to improve OCR. A possible explanation could be a limitation on the availability of additional TCA cycle intermediates, such as acetyl-CoA, whose concentration was likely decreased in rotenone-treated cells due to the preferential use of pyruvate towards lactate production. Oxaloacetate formed after succinate metabolism needs to condense with a molecule of acetyl-CoA for the TCA cycle to function and, consequently, further increase the flux of electrons in the ETC. A further consideration is that since the OCR assay was performed in a short time-frame (approx. 2 h), we cannot exclude that the effect of succinate on OCR could have been more pronounced if cells were exposed longer to succinate. The Seahorse Flux analyser gives an OCR reading per well of cells (mixed glia), and this technology cannot differentiate the individual responses of the various glial sub-types present within the same well. Determining whether there are any differences between the glial sub-types in terms of their OCR in response to rotenone and/or succinate would need dedicated studies of the isolated sub-types.

Succinate yielded a significant decrease in LPR in rotenone-treated cells (Fig. 3D). This concentration-dependent effect remained constant with time, showing a sustained improvement of metabolic function, tested for up to 24 h. Decrease in LPR in succinate-treated wells resulted from a reduction in lactate production and an increase in pyruvate levels compared to cells treated with rotenone only (Fig. 3).

All these metabolic changes occurred in cultures with overall viable cells. Nevertheless, the cell loss that occurred and the changes of cell morphology in cultures treated with rotenone indicates a clear cellular response to the induced metabolic stress.

Our *in vitro* findings are comparable to those *in vivo*. Jalloh *et al*.^11^ tested the hypothesis that 2,3-^13^C_2_ succinate (disodium salt) administered by cerebral microdialysis in TBI patients would be metabolised and enhance the TCA cycle. The measurement of the resulting ^13^C-labelled metabolites, exported from cells into the interstitium and taken up through the microdialysis catheter for analysis, provided definitive proof that the 2,3-^13^C_2_ succinate administered had been metabolised via the TCA cycle^11^. Jalloh *et al*. also reported a lower LPR after focal succinate administration, suggesting an improvement in the mitochondrial function of the cells, even under the metabolically stressful conditions invoked by injury^11^. Use of succinate to support mitochondrial respiration was performed in the presence of adequate oxygenation and not in ischaemia-reperfusion^11^. Our reductionist *in vitro* glial model adds further support to TBI findings *in vivo*.

In conclusion, we have shown that mixed glia cultures respond metabolically to stress induced by rotenone. This is reflected in changes in the LPR and OCR. Moreover, we have shown that succinate is able to improve mitochondria function up to 20% in a sustained manner in mixed glial cultures, consistent with our other studies in succinate-treated focal areas of injured human brain^11^. Our reductionist model of glial mitochondrial stress therefore has potential applicability to devising and evaluating rescue treatments for mitochondrial dysfunction and may pave the way for novel treatments, e.g. administration of succinate, for head injury and for various other conditions associated with mitochondrial dysfunction. Such conditions include neurodegenerative diseases and ageing-related diseases such as dementias and Parkinson’s, which often manifest at a younger age in brain injury survivors compared to individuals without any previous brain injury^24–26^.

## Materials and Methods

### Reagents

Cell culture reagents were purchased from Sigma (Dorset, UK): (Dulbeccos’ Modified Eagle’s Medium (DMEM), L-Glutamine, poly-L-lysine (PLL), papain); or from Life Technologies (Paisley, UK) (fetal bovine serum (FBS), fetal calf serum (FCS), penicillin-streptomycin 100x (Pen-Strep)). All other chemicals were of the purest grade available from Sigma (Dorset, UK) unless otherwise stated.

### Primary mixed glia cell cultures

Primary mixed glia cultures were isolated from P0 to P2 neonatal Sprague-Dawley rat forebrains as previously described^27^. Neonatal rats were euthanized according to “Schedule 1” regulations from the Home Office Animal Procedures Committee UK and the cortices removed. After removing the meninges, tissues were digested using a papain solution and the resulting cell suspensions were seeded in T75 flasks at a density of 2 brains/flask. Cells were cultured in DMEM containing 6 mM glucose and 4 mM glutamine, 1% (v/v) Pen/Strep and 10% (v/v) FBS and medium was changed every 3–4 days. Cells were kept in a humidified incubator at 37 °C under an atmosphere containing 7% CO_2_ in air. After a minimum of 15 days in culture, cells were collected using trypsin-EDTA and re-plated in PLL-coated 6- or 24-well cell culture plates for subsequent experiments at a density of 4.2 × 10^4^ cells/cm^2^.

### Mitochondrial dysfunction experiments

Mitochondrial dysfunction was induced in mixed glial cultures using rotenone, an inhibitor of the complex I of the ETC. When cells reached 80% confluence, the medium was changed to a phenol-red free DMEM (prepared from powdered DMEM from Sigma-Aldrich, cat. no. D5030) containing 6 mM glucose, 1% (v/v) Pen-Strep (final concentrations 100 Units/ml penicillin and 100 µg/ml streptomycin), 1% (v/v) FBS and 0.1, 0.5, 1.0 or 10 µM rotenone and cells were incubated for 24 h. In some wells, succinate at 6, 12 or 24 mM was also added with the aim of assessing its role in improving mitochondrial function in the presence of rotenone. Control wells were incubated with the regular phenol-red free DMEM with or without succinate (6–24 mM). In the 24 h end-point experiments, the medium was collected at the end of the incubation period and saved for metabolite analyses. In the time-course experiments (performed in 6-well plates), samples of medium were collected every 4 h and stored at −20 °C until further analysis. At the end of the experiments, cells were either stained for cell viability assays or washed twice with PBS and fixed for subsequent immunocytochemistry analysis, or lysed for ATP quantification or protein analysis, according to the protocols described below. A minimum of two independent experiments was performed with cells from independent rat litters for each condition tested, using 3 biological replicates per experiment.

### Metabolite Analysis

Samples of culture medium were filtered using 0.45 µm PVDF syringe filter units (Whatman). Quantitative analysis for glucose, lactate and pyruvate was performed using an ISCUSflex microdialysis analyser (M Dialysis AB, Stockholm, Sweden). Two repeat measurements were performed per sample. Data were analysed using M Dialysis LABpilot, Microsoft Excel and OriginLab Origin software.

### Cell viability analysis

Cell viability was assessed in live cells using the cell permeant dye Calcein-AM (Life Technologies, Paisley, UK) (viable cells convert it to Calcein which emits green fluorescence) and propidium iodide (PI) (Life Technologies, Paisley, UK), which enters the nuclei of dead cells, making them fluoresce in red. The dyes were added directly to the cell culture medium: 100 µg/ml of PI solution and 0.6 µM Calcein-AM. After 10 minutes’ incubation at 37 °C, Hoechst 33342 at 5 µg/ml was added to the cells to stain the nuclei. Cells were imaged directly using an IN Cell 2000 Analyser (GE Healthcare, Little Chalfont, UK) in three random fields per well at 20x magnification. The filters used to visualize the different fluorophores were: FITC for calcein, Texas Red for PI and DAPI for Hoescht. The number of dead cells was determined based on the number of PI-positive/Hoechst positive cells and the number of viable cells was given by the ratio Calcein-positive/Hoechst-positive cells. Data are shown as percent of the total number of cells given by Hoechst staining.

### Measurement of cellular oxygen consumption rate (OCR)

Cellular oxygen consumption rate (OCR) was measured using a Seahorse XF24 extracellular flux analyser (Seahorse Biosciences, Denmark). Cells were seeded at a density of 7–10 × 10^4^ cells/well in poly-L-lysine (PLL)-coated XF24 plates and cultured under the conditions described above. The assay was performed when cells reached confluence. Cells were incubated in assay medium (DMEM with all the regular supplements, but minus sodium bicarbonate and phenol red) for at least 30 min prior to the assay under a low CO_2_ environment (XF24 incubator attached to the XF24 analyser). OCR was measured every 5 minutes for three times under basal conditions, after which medium with rotenone, or unsupplemented medium (for the control wells) were added to the culture wells. Three further measurements were then taken before a solution of succinate (disodium salt) at different concentrations (6, 12 and 24 mM) was added to all wells (including controls). In the last step of the assay, 10 μM rotenone + 10 μM antimycin A (inhibitors of mitochondrial complex I and III, respectively) were added to all wells to confirm that the OCR measured was due to mitochondrial respiration (negative control readings). Extracellular acidification rate (ECAR) was also measured on the Seahorse XF24 analyser simultaneously with the OCR measurements in the same wells.

### Quantification of intracellular ATP

Intracellular ATP content was determined using the ATPlite^TM^ 1step kit (Perkin Elmer, Waltham, MA, USA) in lysed cells, according to the manufacturer’s instructions. Luminescence intensity from each well was measured using a GloMax 96 Luminometer microplate reader (Promega, Southampton, UK). Quantification of ATP was made by interpolation from an ATP standard curve made of 10-fold serial dilutions of a 1 µM ATP standard solution. ATP results were normalised by expressing them relative to cellular protein concentration (see below). Data were analysed on Microsoft Excel and OriginLab Origin.

### Protein Assay

Total protein content was determined using the colorimetric Pierce BCA protein assay kit (Thermo Fisher, Scientific, Waltham, MA) in cell pellets, according to the manufacturer’s instructions. Briefly, cells were washed with PBS, collected with trypsin from the cell culture plates and centrifuged to obtain cell pellets, which were then lysed using RIPA lysis buffer (Sigma, Dorset, UK). Absorbance was measured using a Wallac EnVision 2104 Multilabel Reader (Perkin Elmer, Waltham, MA) at 560 nm. Quantification of total protein was determined by interpolation from a standard curve made of 2-fold serial dilutions of a 2 mg/mL bovine serum albumin (BSA) standard solution. Results were calculated using Microsoft Excel and Originlab Origin software.

### Immunocytochemistry

After the mitochondrial dysfunction experiments, cells were fixed with paraformaldehyde (PFA) 4% (v/v) in PBS for 10 min, followed by two washes with PBS. Fixed cells were permeabilized with 0.02% saponin and blocked with 10% normal goat serum prior to incubation with the following primary antibodies overnight at 4 °C: anti-O4 mouse monoclonal antibody (1:1000; R&D Systems, Minneapolis, MN), anti-GFAP rabbit polyclonal antibody (1:500; Dako), anti-GFAP mouse monoclonal antibody (1:300; AbCam, Cambridge, UK), anti-Isolectin-594 conjugated antibody (1:300; Life Technologies), anti-EAAT-1 rabbit polyclonal antibody (1:500; AbCam, Cambridge, UK). Secondary antibodies conjugated with Alexa Fluor 488, Alexa Fluor 594 or Alexa Fluor 647 were used to visualize positive cells (1:500; Life Technologies). The nuclei were stained using DAPI (Life Technologies). Following immunocytochemistry, cells were mounted with Prolong gold anti-fade mounting medium (Life Technologies). Cells were visualised and images acquired using a Zeiss LSM 700 confocal microscope (Zeiss, Thornwood, NJ) at 20x magnification using the Zen Application software. For cells stained directly on cell culture plates, images from three random fields were acquired per well using an IN Cell Analyser 2000 (GE Healthcare, Little Chalfont, UK) microscope at 20x magnification. For quantitative analyses, the percentage positive cells for a given antigen relative to >200 DAPI-stained nuclei per experiment was determined in randomly selected visual fields.

### Statistical analysis

Data are shown as mean ± S.E.M. values from samples obtained from at least two independent experiments or, for time-course experiments, data from one representative experiment is provided, with statistics performed for the biological replicates used (n = 3). Data were analysed by two-way ANOVA followed by Tukey’s means comparison post-hoc test (to compare rotenone treated with control samples), or using Student’s paired T-test to compare the effect of succinate vs non-succinate. Calculations were performed using the statistical software package OriginLab Origin. Differences were considered significant when p < 0.05.

## References

[1] Phang I, Zoumprouli A, Papadopoulos MC, Saadoun S (2016). Microdialysis to optimize cord perfusion and drug delivery in spinal cord injury. Ann Neurol.

[2] Okon EB (2013). Intraparenchymal Microdialysis after Acute Spinal Cord Injury Reveals Differential Metabolic Responses to Contusive versus Compressive Mechanisms of Injury. Journal of neurotrauma.

[3] Timofeev I (2011). Cerebral extracellular chemistry and outcome following traumatic brain injury: a microdialysis study of 223 patients. Brain.

[4] Timofeev I (2011). Interaction between brain chemistry and physiology after traumatic brain injury: impact of autoregulation and microdialysis catheter location. Journal of neurotrauma.

[5] Vespa P (2005). Metabolic crisis without brain ischemia is common after traumatic brain injury: a combined microdialysis and positron emission tomography study. Journal of Cerebral Blood Flow & Metabolism.

[6] Helmy A, Vizcaychipi M, Gupta A (2007). Traumatic brain injury: intensive care management. Brit J Anaesth.

[7] Nordström C-H, Nielsen TH, Schalén W, Reinstrup P, Ungerstedt U (2016). Biochemical indications of cerebral ischaemia and mitochondrial dysfunction in severe brain trauma analysed with regard to type of lesion. Acta neurochirurgica.

[8] Verweij BH (2000). Impaired cerebral mitochondrial function after traumatic brain injury in humans. Journal of neurosurgery.

[9] McCormack JG, Halestrap AP, Denton RM (1990). Role of calcium ions in regulation of mammalian intramitochondrial metabolism. Physiological reviews.

[10] Cheng G, Kong Rh, Zhang Lm, Zhang Jn (2012). Mitochondria in traumatic brain injury and mitochondrial‐targeted multipotential therapeutic strategies. British journal of pharmacology.

[11] Jalloh, I. *et al*. Focally perfused succinate potentiates brain metabolism in head injury patients. *Journal of Cerebral Blood Flow & Metabolism*, 0271678X16672665, doi:10.1177/0271678X16672665 (2016). 10.1177/0271678X16672665 PMC5482384 27798266

[12] Rich PR, Maréchal A (2010). The mitochondrial respiratory chain. Essays in biochemistry.

[13] Berg, J. M., Tymoczko, J. L. & Stryer, L. Biochemistry, 5th Ed. (W. H. Freeman, 2002).

[14] Protti A, Singer M (2006). Bench-to-bedside review: potential strategies to protect or reverse mitochondrial dysfunction in sepsis-induced organ failure. Critical Care.

[15] Eleff S (1984). 31P NMR study of improvement in oxidative phosphorylation by vitamins K3 and C in a patient with a defect in electron transport at complex III in skeletal muscle. Proceedings of the National Academy of Sciences.

[16] Ferreira F, Ladriere L, Vincent J-L, Malaisse W (2000). Prolongation of survival time by infusion of succinic acid dimethyl ester in a caecal ligation and perforation model of sepsis. Hormone and Metabolic Research.

[17] Ehinger JK (2016). Cell-permeable succinate prodrugs bypass mitochondrial complex I deficiency. Nature communications.

[18] Divakaruni A, Paradyse A, Ferrick D, Murphy A, Jastroch M (2013). Analysis and interpretation of microplate-based oxygen consumption and pH data. Methods in enzymology.

[19] Glick D, Barth S, Macleod KF (2010). Autophagy: cellular and molecular mechanisms. The Journal of pathology.

[20] Falkowska A (2015). Energy metabolism of the brain, including the cooperation between astrocytes and neurons, especially in the context of glycogen metabolism. Int J Mol Sci.

[21] Hertz L, Peng L, Dienel GA (2007). Energy metabolism in astrocytes: high rate of oxidative metabolism and spatiotemporal dependence on glycolysis/glycogenolysis. Journal of Cerebral Blood Flow & Metabolism.

[22] Hertz L (2015). Astrocytic glycogenolysis: mechanisms and functions. Metabolic brain disease.

[23] Hillered L, Persson L, Nilsson P, Ronne-Engstrom E, Enblad P (2006). Continuous monitoring of cerebral metabolism in traumatic brain injury: a focus on cerebral microdialysis. Current opinion in critical care.

[24] Gardner RC (2014). Dementia risk after traumatic brain injury vs nonbrain trauma: the role of age and severity. JAMA neurology.

[25] Gardner RC (2015). Traumatic brain injury in later life increases risk for Parkinson disease. Ann Neurol.

[26] Perry DC (2016). Association of traumatic brain injury with subsequent neurological and psychiatric disease: a meta-analysis. Journal of neurosurgery.

[27] Amaral AI, Hadera MG, Tavares JM, Kotter M, Sonnewald U (2016). Characterization of glucose‐related metabolic pathways in differentiated rat oligodendrocyte lineage cells. Glia.

